# Chaos Unleashed: The impact of recreational drugs and COVID on young adults

**DOI:** 10.1192/j.eurpsy.2022.618

**Published:** 2022-09-01

**Authors:** A. Makela, M. Howard

**Affiliations:** 1 Cambridge Health Alliance/Harvard Medical School, Department Of Psychiatry, Cambridge, United States of America; 2 Harvard Medical School at Cambridge Health Alliance, Department Of Psychiatry, Cambridge, United States of America

**Keywords:** drug use, case series, young adults, schizoaffective

## Abstract

**Introduction:**

This case series reveals a number of young adults, whom after chronic use of recreational drugs, suffer the life-long consequence of severe chronic mental illness.

**Objectives:**

• Review the illicit drugs that are commonly associated with psychotic symptoms. • Highlight exposures theorized to impact genetics associated with DSM 5 diseases. • Compare trends in illicit drug use during the worldwide COVID pandemic.

**Methods:**

A literature review is used to examine the impact of COVID pandemic on illicit drug use in metropolitan cities in European countries and compare the trends with what is seen by the consult liaison psychiatry service at a metropolitan community hospital in the USA.

**Results:**

In European Countries with data available, there were measurable differences in which illicit drugs were used most during the COVID 19 pandemic. In the US this data is not readily available at the time of submission for proper comparisson.

**Conclusions:**

Although definitive comparrison is pending, the results of extensive illicit drug use demostrate a high comorbidity with psychotic spectrum disorders in the DSM 5.

**Disclosure:**

No significant relationships.

